# Declining incidence of cerebral palsy in South Korea

**DOI:** 10.1038/s41598-023-36236-8

**Published:** 2023-06-28

**Authors:** Gun-Ha Kim, Gisu Lee, Sungyeon Ha, Geum Joon Cho, Yoon Ha Kim

**Affiliations:** 1 Department of Pediatrics, Korea Cancer Center Hospital, Korea Institute of Radiological and Medical Sciences, Seoul, Korea; 2 Department of Obstetrics and Gynecology, Keimyung University School of Medicine, Daegu, Korea; 3 Graduate School of Statistics, Sungkyunkwan University, Seoul, Korea; 4 Department of Obstetrics and Gynecology, Korea University Guro Hospital, Korea University College of Medicine, 148 Gurodong-Ro, Guro-Gu, Seoul, 08308 Korea; 5 Department of Obstetrics and Gynecology, Chonnam National University Medical School, 160 Baekseo-Ro, Dong-Gu, Gwangju, 61469 Korea

**Keywords:** Diseases, Health care, Medical research, Neurology, Risk factors

## Abstract

Presuming that the incidence of cerebral palsy (CP) in Korea is decreasing due to medical advances, we analyzed the trends and risk factors of CP in changing circumstances. We identified all women who delivered a singleton between 2007 and 2015 using the Korea National Health Insurance (KNHI). Information on pregnancy and birth was obtained by linking the KNHI claims database and data from the national health-screening program for infants and children. The 4-years incidence of CP decreased significantly from 4.77 to 2.52 per 1000 babies during the study period. The multivariate analysis revealed that the risk of developing CP was 29.5 times higher in preterm infants born before 28 weeks of gestational age, 24.5 times higher in infants born between 28 and 34 weeks, and 4.5 times higher in infants born between 34 and 36 weeks, compared to full-term appropriate for age (2.5 ~ 4 kg of body weight) infants. 5.6 times higher in those with birth weight < 2500 g, and 3.8 times higher in pregnancies with polyhydramnios. Additionally, respiratory distress syndrome increased the risk of developing CP by 2.04 times, while necrotizing enterocolitis was associated with a 2.80-fold increased risk of CP. In Korea, the incidence of CP in singleton decreased from 2007 to 2015. We need to continue to focus on developing medical technologies for the early detection of high-risk neonates and minimizing brain damage to reduce the incidence rate of CP effectively.

## Introduction

Cerebral palsy (CP) is a group of permanent disorders of the development of movement and posture causing activity limitation attributed to non-progressive disturbances in the developing fetal or infant brain^1^. In addition to physical disability, CP can be accompanied by cognitive impairment, communication problems, and epilepsy, which cause considerable personal and socioeconomic burdens. The medical community’s ongoing efforts to reduce this burden have significantly improved healthcare technology over the past few decades. The early identification of women at risk of preterm birth and protective methods for reducing perinatal complications may decrease the incidence of preterm birth and fetal mortality^2^. Moreover, asphyxia is suspected, brain cooling has been widely used to protect the neonatal brain. Presuming that the incidence of CP in Korea is decreasing due to medical advances, we attempted to analyze the latest trends and risk factors for CP in changing circumstances.

## Methods

### Healthcare delivery system in Korea

Almost all Koreans are covered by the health insurance policies of the Korea National Health Insurance (KNHI) of the Health Insurance Review and Assessment Service, except for 3% of the population that the Medical Aid Program covers. Thus, the KNHI claims database contains claims information for most Koreans except for uninsured procedures, such as plastic surgery. As a part of the National Health Insurance Corporation healthcare system, the national health-screening program for infants and children, for which children aged 4–80 months are eligible, began in 2007 and is composed of seven consecutive health examinations according to age (4–9, 9–18, 18–30, 30–42, 42–54, 54–66, and 66–80 months). The national health-screening program consists of history taking, physical examination, developmental screening, visual acuity, and a dental examination.

### Sample and study design

Information on pregnancy and birth was obtained by linking the KNHI claims database and national health screening program data for infants and children. Using the KNHI claims database, we identified all women who delivered babies between 2007 and 2015. A total of 3,778,561 women who had deliveries were identified. Excluding 56,925 deliveries of multiple births, a total of 3,278,916 single births were included in our study. To ensure the reliability and validity of our analysis, we excluded twin babies from the study sample as they could result in duplicate data from the same mothers. Moreover, since twin births represent only a small proportion of all births, their exclusion allowed for a more representative sample of the general population. Detailed information, such as medical illness, delivery mode, pregnancy complications, and single or multiple pregnancies, was confirmed using data from the KNHI claims and the national health-screening program for infants and children.

### CP case definition

We assumed an initial diagnosis of CP for early treatment if a diagnostic code had been registered. In addition, we defined cases of cerebral palsy using the Tenth Revision of the International Classification of Diseases: spastic quadriplegic CP (G80.0), spastic diplegic CP (G80.1), spastic hemiplegic CP (G80.2), dyskinetic CP (G80.3), ataxic CP (G80.4), other CP (G80.8), CP, unspecified (G80.9). Hereditary spastic paraplegia (G11.4) was excluded. Upon diagnosis of CP, the number of people was summed based on the child's birth year. The observational period was at least four years after birth.

### Incidence of cerebral palsy

The yearly incidence of CP per 1000 live births was calculated using the total number of infants born during the study year as the denominator and the number of infants diagnosed with CP during subsequent follow-up as the numerator.

### Risk variables

1. Pre-pregnancy health status: “hypertension (I10)”, “diabetes mellitus (E11)”, and “advanced maternal age (> 35 years)”.
2. Pregnancy complications: “gestational hypertension (O13)”, “preeclampsia (O14)”, “gestational diabetes (O24)”, “chorioamnionitis (O41.1)”, “premature rupture of membrane (O42)”, “oligohydramnios (O41.0)”, “polyhydramnios (O40)”.
3. Conditions during delivery: “preterm birth (< 37 weeks of gestation)”, “mode of delivery”, “low birth weight (< 2500 g)”, and “large for gestational age (≥ 4000 g)”.
4. Conditions after delivery: “respiratory distress syndrome (P22.0)”, “Bronchopulmonary dysplasia (P27.1)”, “Necrotizing enterocolitis (K55.3)”.

### Statistical analysis

Statistical analysis was performed using SPSS software version 12.0 (SPSS Inc., Chicago, IL, USA). The Student's t-test was used to compare continuous variables between groups. The categorical variables were compared using the χ2 test. We compared temporal trends by using the χ2 test. Multivariate logistic regression analysis was used to estimate the adjusted odds ratio (OR) and 95% confidence interval (CI). All P-values were two-sided and were considered statistically significant if 0.050 or less.

### Ethics approval and consent to participate

The institutional review board of the Korea University Guro Hospital approved the study (2020GR0468) and granted a waiver for informed consent because of its retrospective nature.

## Results

### Trends in vaginal and cesarean births by year

**Figure 1:**
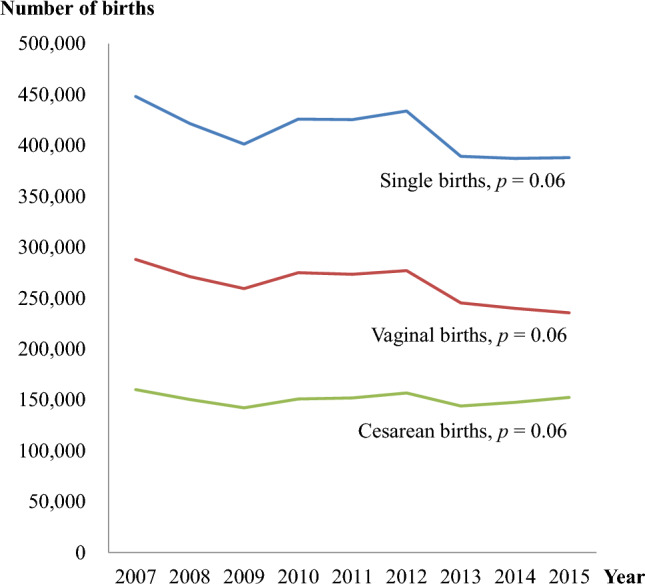
Trends in the number of single births and delivery methods, 2007–2015.

As shown in Fig. 1, the number of single vaginal births from 2007 to 2015 appeared to be modestly declining, while the number of cesarean deliveries increased slightly, though the changes were insignificant.

### Incidence of cerebral palsy

**Figure 2:**
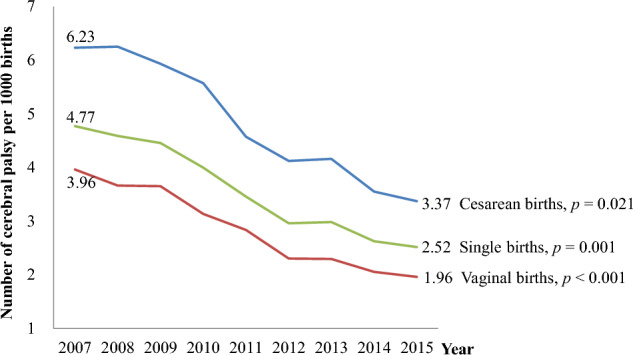
Changes in the incidence of cerebral palsy in children born between 2007 and 2015. Cerebral palsy per 1000 live births declined yearly and has nearly halved over the past eight years.

As shown in Fig. 2, the incidence of CP decreased from 4.77 to 2.52 per 1000 single live births, from 6.23 to 3.37 among cesarean deliveries, and from 3.96 to 1.96 among vaginal births (*p* < 0.001 for vaginal births; *p *= 0.001 for total live births; *p* = 0.021 for cesarean births).

### Comparison of the study population

According to a combined analysis of maternal and their babies' records, the frequency of advanced maternal age, hypertension, and diabetes before and during pregnancy, diabetes mellitus before pregnancy, cesarean delivery, gestational hypertension, gestational diabetes mellitus requiring insulin treatment, chorioamnionitis, premature rupture of membrane, oligo- and polyhydramnios, preterm birth, low birth weight, male sex, and large for gestational age was higher in the group diagnosed with CP than in the controls (Table 1).

**Table 1:** Comparison of study populations.

	Control (n = 3,268,693)	Cerebral palsy (n = 10,223)	*P*-value
Maternal age (years)	30.876 ± 3.87	30.9997 ± 4.07	0.002
Maternal age > 35 years	538,542 (16.48%)	1871 (18.3%)	< 0.001
Hypertension	106,401 (3.26%)	500 (4.89%)	< 0.001
DM	156,421 (4.79%)	647 (6.33%)	< 0.001
Cesarean delivery	1,178,746 (36.06%)	4873 (47.67%)	< 0.001
Gestational hypertension	62,801 (1.92%)	643 (6.29%)	< 0.001
GDM without insulin	276,871 (8.47%)	834 (8.16%)	0.258
GDM with insulin	25,193 (0.77%)	165 (1.61%)	< 0.001
Chorioamnionitis	23,195 (0.71%)	266 (2.6%)	< 0.001
Premature rupture of membrane	518,472 (15.86%)	2327 (22.76%)	< 0.001
Oligohydramnios	34,660 (1.06%)	356 (3.48%)	< 0.001
Polyhydramnios	3394 (0.1%)	88 (0.86%)	< 0.001
Preterm birth (< 37 weeks)			< 0.001
< 28 weeks	12,452 (0.38%)	838 (8.20%)	
28–33/6 weeks	22,400 (0.69%)	1457 (14.25%)	
36/6 weeks	57,472 (1.76%)	631 (6.17%)	
Term birth (≥ 37 weeks)	3,176,369 (97.18%)	7297 (71.38%)	< 0.001
With BW < 2500 g	62,148 (1.9%)	747 (7.31%)	
With 2.5 kg ≤ BW < 4.0 kg	2,990,361 (91.48%)	6307 (61.69%)	
With BW ≥ 4.0 kg	123,860 (3.79%)	243 (2.38%)	
Male	1,683,526 (51.5%)	5984 (58.53%)	< 0.001
Respiratory distress syndrome	6681(0.2%)	140(1.37%)	< 0.001
Bronchopulmonary dysplasia	76(0%)	2(0.02%)	0.0004
Necrotizing enterocolitis	148(0%)	5(0.05%)	< 0.001

Statistical analyses were performed using the t-test and χ2 test. Values are presented as mean (standard deviation) or n (%). Significant if *P* < 0.05.

Abbreviations: *DM* diabetes mellitus, *GDM* gestational diabetes mellitus, *BW* body weight.

### Risk factors for developing cerebral palsy

In Table 2, the multivariate analysis revealed that gestational age and other factors significantly impact the risk of CP in infants. Most of all, babies born before 28 weeks of gestational age have the highest risk, with a 29.49 times higher likelihood of developing CP than full-term babies of the appropriate weight. Babies born between 28 and 34 weeks and between 34 and 36 weeks have a 24.55 and 4.54 times higher risk, respectively, compared to full-term babies of the appropriate weight. Full-term babies who are small-for-gestational-age (body weight < 2.5kg) have a 5.39 times higher risk of developing CP compared to full-term babies of appropriate weight, and pregnancies with polyhydramnios have a 3.81 times higher risk of CP. Additionally, respiratory distress syndrome increased the risk of developing CP by 2.04 times, while necrotizing enterocolitis was associated with a 2.80-fold increased risk of CP.

**Table 2:** Risk factors for developing cerebral palsy in singleton pregnancy.

	Unadjusted OR (95% CI)	Adjusted OR (95% CI)
Maternal age > 35 years	1.14 (1.08–1.19)	0.99 (0.94–1.04)
HTN	1.53 (1.40–1.67)	1.11 (1.01–1.22)
DM	1.35 (1.24–1.46)	1.15 (1.06–1.25)
Cesarean delivery	1.62 (1.55–1.68)	1.33 (1.27–1.38)
Preeclampsia	3.43 (3.16–3.71)	1.18 (1.08–1.28)
GDM without insulin	0.96 (0.90–1.03)	0.83 (0.77–0.90)
GDM with insulin	2.12 (1.81–2.47)	1.47 (1.23–1.75)
Chorioamnionitis	3.74 (3.31–4.23)	1.54 (1.36–1.75)
Preterm rupture of membrane	1.56 (1.49–1.64)	1.13 (1.07–1.18)
Oligohydramnios	3.37 (3.03–3.74)	1.67 (1.50–1.87)
Polyhydramnios	8.37 (6.77–10.35)	3.81 (3.04–4.77)
Preterm birth		
< 28 weeks	31.91 (29.63–34.37)	29.49 (27.35–31.79)
28–33/6 weeks	30.84 (29.09–32.70)	24.55 (23.00–26.20)
34–36/6 weeks	5.21 (4.80–5.65)	4.54 (4.18–4.94)
Term birth with BW < 2500 g	5.70 (5.28–6.15)	5.39 (4.99–5.82)
With 2.5 kg ≤ BW < 4.0 kg	1	1
With BW ≥ 4.0 kg	0.93 (0.82–1.06)	0.86 (0.76–0.98)
Male	1.33 (1.28–1.38)	1.32 (1.27–1.38)
Respiratory distress syndrome	6.78 (5.73–8.03)	2.04 (1.69–2.47)
Bronchopulmonary dysplasia	8.58 (2.13–34.49)	1.27 (0.31–5.24)
Necrotizing enterocolitis	10.81 (4.43–26.36)	2.80 (1.14–6.87)

Multivariate logistic regression analysis was used to estimate the adjusted odds ratio (OR) and 95% confidence interval (CI). BW, body weight. Adjusted for variables in the table.

### The annual incidence rate of risk factors

**Figure 3:**
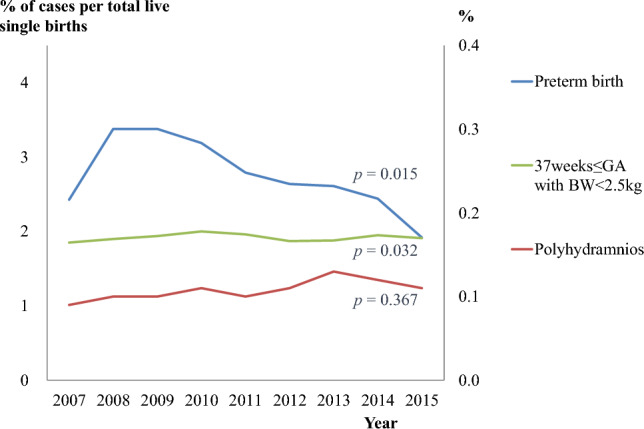
Annual trends in risk factor occurrence. There was a slight decrease in the percentage of preterm births, whereas the proportion of full-term small-for-gestational-age increased slightly. However, there was no significant change in the frequency of polyhydramnios throughout the study period.

Figure 3 presents the annual incidence rate of cerebral palsy's three major risk factors. Our results demonstrate a statistically significant reduction in the incidence of preterm birth, from 3.23 to 1.91% (*p * = 0.015), as well as a slight increase in the proportion of full-term small-for-gestational-age, from 1.85 to 1.91% (*p * = 0.032). On the other hand, we observed no significant variation in the occurrence rate of polyhydramnios over the study period.

## Discussion

Our data revealed that the incidence of CP per 1000 live births declined significantly from 4.77 to 2.52 babies among all live single births between 2007 and 2015. The likelihood of developing CP is 4.54–29.49 times higher in preterm babies than in full-term babies of the appropriate weight. Additionally, full-term babies who are small-for-gestational-age (body weight <2.5kg) have a 5.39 times higher risk of developing CP, and pregnancies with polyhydramnios have a 3.81 times higher risk of CP. Neonatal conditions, such as respiratory distress syndrome and necrotizing enterocolitis, have increased the risk of developing CP by 2.04-fold and 2.80-fold, respectively.

### The incidence rate of cerebral palsy

A previous study in South Korea found that the incidence of CP increased from 2.2 to 3.2 per 1000 children born between 1999 and 2003^3^. According to a meta-analysis analyzing 19 previous reports using live births as the denominator, the overall prevalence of CP is 2.11 per 1000 births^4^. The included studies were from the USA, Canada, Australia, or Europe, except one from China. Nationwide population studies in other Asian countries have recently reported a prevalence of CP of 1.09 per 1000 children under 20 years of age in Taiwan^5^, 1.19 in children aged 0–6 years in China^6^, and 2.26 in children aged 0–4 years in Japan^7^. Our study findings indicate that the incidence of CP in Korea experienced a significant decline from 4.77 to 2.52 per 1000 live births from 2007 to 2015. This decrease was similar to the degree observed in other Asian countries.

### Risk factors for cerebral palsy

#### Preterm birth

Aside from our study results, preterm birth is already well-known as a risk factor for CP^8,9^. The immature brain cannot effectively maintain a blood supply because there are fewer collateral vessels or anastomoses around the peripheral blood vessels and immature walls. The vessels cannot compensate for hypoxic-ischemic damage to limited vasodilation capacity^10^. Thus, various attempts have been made to prevent preterm birth; 17α-hydroxyprogesterone caproate may help prevent recurrent preterm birth^11–13^. Furthermore, vaginal progesterone prevents premature birth of mothers with short cervixes and improves neonatal outcomes^14–16^. Overall, progesterone administration lowered the rate of preterm births by approximately 50%^15,17^. Cervical pessary use or cervical cerclage prevents repeated preterm births in high-risk women with a short cervix^18^. Despite medical advances, the rate of preterm births increased from 2.9% (1997–1999) to 4.5% (2011–2013)^19^ and from 3.31% (1997–1998) to 6.44% (2013–2014)^20^ in Korea. The increase in twin births is thought to have contributed to the rise in preterm births. As shown in Fig. 3, singleton's incidence of preterm birth slightly decreased between 2007 and 2015 (*p *= 0.015) in our study.

#### Fetal growth restriction

The association between the development of CP and fetal growth restriction is well known^21^. Fetal growth restriction has been reported as a more critical risk factor for CP than fetal inflammation and birth asphyxia combined^22^. In our study, based on the limitations of the available data, an alternative approach was taken to analyze the risk of cerebral palsy associated with growth abnormalities. Since accurate gestational age data was unavailable from the claim data, term babies were categorized based on their birth weight into three groups: those weighing less than 2.5kg, those weighing between 2.5kg and 4kg, and those weighing 4kg or more. Despite the limitations, this method allowed for a more detailed investigation of the potential risk factors linked to cerebral palsy in infants with growth abnormalities. Our results showed that full-term babies who are small-for-gestational-age (body weight <2.5kg) have a 5.39 times higher risk of developing CP compared to full-term babies of appropriate weight, concordant with the previous studies.

#### Polyhydramnios

In our study, polyhydramnios increased the risk of CP by 3.81 times. Polyhydramnios has also been associated with increased perinatal morbidity and mortality risk, such as preterm birth, aneuploidy, cesarean section, fetal anomalies, and perinatal and postnatal mortality^23,24^. Even when the results of a detailed ultrasound examination of the fetus were normal, polyhydramnios doubled the risk of genetic syndromes, neurologic disorders, and fetal malformations diagnosed after birth^25^. The incidence of polyhydramnios did not change over the study period (Fig. 3, *p *= 0.367).

#### Postnatal risks

Our study found that respiratory distress syndrome and necrotizing enterocolitis were associated with a 2.04-fold and 2.80-fold increased risk of developing CP, respectively. Our study's results align with the prior studies, as it reported that moderately late and late preterm infants (32–36 weeks) who experienced respiratory distress syndrome had a two times higher incidence of CP than those without respiratory distress syndrome at the same gestational weeks^26^. Moreover, a meta-analysis demonstrated a 1.59-fold increased risk of CP in neonates with necrotizing enterocolitis, possibly due to heightened exposure to proinflammatory cytokines and the associated risk of sepsis^27^.

In addition, it is important to note that although not included in our study data, the advancements in various medical technologies aimed at protecting the neonatal brain may have contributed to a reduction in the incidence rate of CP. First, magnesium sulfate (MgSO_4_) stabilizes blood pressure, reduces vasoconstriction in the cerebral arteries, and restores circulation in preterm neonates^28,29^. Treatment with MgSO_4_ in preterm labor may lower the risk of CP^30–32^. The proportion of moderate to severe CP decreased significantly in babies born in women with preterm birth who were treated with magnesium (relative risk, 0.55; 95% CI 0.32–0.95)^31^; several meta-analyses also support this result^33–35^. Second, the prenatal administration of corticosteroids for fetal lung maturity may reduce the occurrence of CP^36^. Finally, brain or whole-body cooling has become standard management for neuroprotection in newborns with birth asphyxia^37,38^.

Through various efforts, including improvements in prenatal and neonatal care, Korea has decreased the infant mortality rate from 4.7 in 2004 to 3.0 in 2014^39^. Moreover, our study revealed a continued decline in the incidence of CP in Korea.

### Limitations

In this study, we assumed that the registration of a diagnostic code indicates an initial diagnosis of CP. However, it is important to acknowledge that diagnosing CP can be challenging and may change as more information is gathered or the child develops. Children with mild CP may not be diagnosed until later in life, as their symptoms may not be as apparent during early childhood. Therefore, our study may include cases where a tentative diagnosis was made to receive early physical therapy. In some healthcare settings, it is common practice for healthcare professionals to diagnose a child with CP for this purpose tentatively. However, it is important to note that including such cases may have contributed to potential inaccuracies in our findings, which should be considered when interpreting our results. We also acknowledge the limitations of relying solely on diagnostic codes for diagnosing CP in our study. Using administrative data in research may result in potential inaccuracies due to the lack of clinical data in the analysis, a recognized limitation commonly encountered in studies utilizing administrative data. Given the absence of accurate gestational age data in the medical claim database, we categorized term babies based on birth weight and analyzed them to alternatively assess the risk of cerebral palsy associated with growth abnormalities.

While our study successfully linked birth records of infants born in the hospital to their respective mothers, it is essential to note that our study's scope is limited to infants born within the hospital. Thus, our study did not include roughly 1.5% of births outside the hospital. Furthermore, a small number of individuals who may have emigrated or died at some point after birth cannot verify their status within our research data.

Finally, while a child can be diagnosed with CP if a triggering event occurs before the affected function fully develops^1^, our study was unable to identify perinatal risk factors of CP such as birth asphyxia, neonatal sepsis, or respiratory distress syndrome, as well as early infantile risks such as encephalitis or head trauma.

## Conclusion

In Korea, the incidence of CP decreased from 2007 to 2015. Based on our findings and analysis, we can infer that the efforts to protect the brain of neonates before and after birth through advanced technologies have effectively reduced the incidence rate of CP. Therefore, we need to continue to focus on developing medical technologies for the early detection of high-risk neonates and minimizing brain damage to reduce the incidence rate of CP effectively.

## Data Availability

The datasets used or analyzed during the current study are available from the corresponding author upon reasonable request.

## References

[1] Rosenbaum P (2007). A report: The definition and classification of cerebral palsy April 2006. Dev. Med. Child Neurol. Suppl..

[2] Jung EJ (2018). Antenatal magnesium sulfate for both tocolysis and fetal neuroprotection in premature rupture of the membranes before 32 weeks’ gestation. J. Maternal-Fetal Neonatal Med..

[3] Park MS (2011). Prevalence and lifetime healthcare cost of cerebral palsy in South Korea. Health Policy.

[4] Oskoui M (2013). An update on the prevalence of cerebral palsy: A systematic review and meta-analysis. Dev. Med. Child Neurol..

[5] Chiang K-L, Kuo F-C, Cheng C-Y, Chang K-P (2019). Prevalence and demographic characteristics of comorbid epilepsy in children and adolescents with cerebral palsy: A nationwide population-based study. Childs Nerv. Syst..

[6] He P (2017). Children with motor impairment related to cerebral palsy: Prevalence, severity and concurrent impairments in China. J. Paediatr. Child Health.

[7] Toyokawa S, Maeda E, Kobayashi YJDM, Neurology C (2017). Estimation of the number of children with cerebral palsy using nationwide health insurance claims data in Japan. Dev. Med. Child Neurol..

[8] Vincer MJ (2006). Increasing prevalence of cerebral palsy among very preterm infants: A population-based study. Pediatrics.

[9] Ancel P-Y (2006). Cerebral palsy among very preterm children in relation to gestational age and neonatal ultrasound abnormalities: The EPIPAGE cohort study. Pediatrics.

[10] Bauer R (1991). Interaction between systemic circulation and brain injuries in newborns. Exp. Pathol..

[11] Johnson JW, Austin KL, Jones GS, Davis GH, King TM (1975). Efficacy of 17α-hydroxyprogesterone caproate in the prevention of premature labor. N. Engl. J. Med..

[12] Yemini M (1985). Prevention of premature labor by 17α-hydroxyprogesterone caproate. Am. J. Obstet. Gynecol..

[13] Meis PJ (2003). Prevention of recurrent preterm delivery by 17 alpha-hydroxyprogesterone caproate. N. Engl. J. Med..

[14] De Franco E (2007). Vaginal progesterone is associated with a decrease in risk for early preterm birth and improved neonatal outcome in women with a short cervix: A secondary analysis from a randomized, double-blind, placebo-controlled trial. Ultrasound Obst. Gynecol..

[15] Hassan S (2011). Vaginal progesterone reduces the rate of preterm birth in women with a sonographic short cervix: A multicenter, randomized, double-blind, placebo-controlled trial. Ultrasound Obst. Gynecol..

[16] da Fonseca EB, Bittar RE, Carvalho MH, Zugaib MJ (2003). Prophylactic administration of progesterone by vaginal suppository to reduce the incidence of spontaneous preterm birth in women at increased risk: A randomized placebo-controlled double-blind study. Am. J. Obstet. Gynecol..

[17] Romero R (2012). Vaginal progesterone in women with an asymptomatic sonographic short cervix in the midtrimester decreases preterm delivery and neonatal morbidity: A systematic review and metaanalysis of individual patient data. Am. J. Obst. Gynecol..

[18] Goya M (2012). Cervical pessary in pregnant women with a short cervix (PECEP): An open-label randomised controlled trial. Lancet.

[19] Park SH, Lim DO (2016). Secular trends of gestational length distribution in Korean Singleton and twin birth: 1997–1999, 2011–2013. J. Korean Soc. Matern. Child Health.

[20] Park SH, Kim JS, Lim DO (2017). Secular trend of gestational age specific preterm birth rate in Korean singleton and multiple birth: 1997–1998, 2013–2014. J. Health Inf. Stat..

[21] Croen LA, Grether JK, Curry CJ, Nelson KB (2001). Congenital abnormalities among children with cerebral palsy: More evidence for prenatal antecedents. J. Pediatr..

[22] McIntyre S (2013). Antecedents of cerebral palsy and perinatal death in term and late preterm singletons. Obstet. Gynecol..

[23] Pauer H-U (2003). Incidence of fetal malformations in pregnancies complicated by oligo-and polyhydramnios. Arch. Gynecol. Obstet..

[24] Morris R (2014). Association and prediction of amniotic fluid measurements for adverse pregnancy outcome: Systematic review and meta-analysis. BJOG Int. J. Obstet. Gynaecol..

[25] Yefet E, Daniel-Spiegel EJP (2016). Outcomes from polyhydramnios with normal ultrasound. Pediatrics.

[26] Thygesen SK, Olsen M, Østergaard JR, Sørensen HT (2016). Respiratory distress syndrome in moderately late and late preterm infants and risk of cerebral palsy: A population-based cohort study. BMJ Open.

[27] Schulzke SM, Deshpande GC, Patole SK (2007). Neurodevelopmental outcomes of very low-birth-weight infants with necrotizing enterocolitis: A systematic review of observational studies. Arch. Pediatr. Adolesc. Med..

[28] de Haan HH, Gunn AJ, Williams CE, Heymann MA, Gluckman PD (1997). Magnesium sulfate therapy during asphyxia in near-term fetal lambs does not compromise the fetus but does not reduce cerebral injury. Am. J. Obstet. Gynecol..

[29] Schiff SJ, Somjen GG (1985). Hyperexcitability following moderate hypoxia in hippocampal tissue slices. Brain Res..

[30] Nelson KB, Grether JK (1995). Can magnesium sulfate reduce the risk of cerebral palsy in very low birthweight infants?. Pediatrics.

[31] Rouse DJ (2008). A randomized, controlled trial of magnesium sulfate for the prevention of cerebral palsy. N. Engl. J. Med..

[32] Gibbins KJ (2013). Evaluation of the clinical use of magnesium sulfate for cerebral palsy prevention. Obstet. Gynecol..

[33] Conde-Agudelo A, Romero R (2009). Antenatal magnesium sulfate for the prevention of cerebral palsy in preterm infants less than 34 weeks' gestation: a systematic review and metaanalysis. Am. J. Obstet. Gynecol..

[34] Costantine MM, Weiner SJ (2009). Effects of antenatal exposure to magnesium sulfate on neuroprotection and mortality in preterm infants: a meta-analysis. Obstet. Gynecol..

[35] Doyle, L. W., Crowther, C. A., Middleton, P., Marret, S. & Rouse, D. J. Magnesium sulphate for women at risk of preterm birth for neuroprotection of the fetus. *Cochrane Datab. Syst. Rev.* (2009). 10.1002/14651858.CD004661.pub3 19160238

[36] Sotiriadis A (2015). Neurodevelopmental outcome after a single course of antenatal steroids in children born preterm: a systematic review and meta-analysis. Obstet. Gynecol..

[37] Azzopardi DV (2009). Moderate hypothermia to treat perinatal asphyxial encephalopathy. N. Engl. J. Med..

[38] Edwards A, Azzopardi DV (2006). Therapeutic hypothermia following perinatal asphyxia. Arch. Dis. Childhood-Fetal Neonatal. Ed..

[39] Shin H-Y (2017). Infant, maternal, and perinatal mortality statistics in the Republic of Korea, 2014. J. Korean Med. Assoc..

